# Numerical Investigation of Pulse Wave Propagation in Arteries Using Fluid Structure Interaction Capabilities

**DOI:** 10.1155/2017/4198095

**Published:** 2017-09-24

**Authors:** Hisham Elkenani, Essam Al-Bahkali, Mhamed Souli

**Affiliations:** ^1^Department of Mechanical Engineering, King Saud University, P.O. Box 800, Riyadh 11421, Saudi Arabia; ^2^Laboratoire de Mécanique de Lille, UMR CNRS 8107, Villeneuve-d'Ascq, France

## Abstract

The aim of this study is to present a reliable computational scheme to serve in pulse wave velocity (PWV) assessment in large arteries. Clinicians considered it as an indication of human blood vessels' stiffness. The simulation of PWV was conducted using a 3D elastic tube representing an artery. The constitutive material model specific for vascular applications was applied to the tube material. The fluid was defined with an equation of state representing the blood material. The onset of a velocity pulse was applied at the tube inlet to produce wave propagation. The Coupled Eulerian-Lagrangian (CEL) modeling technique with fluid structure interaction (FSI) was implemented. The scaling of sound speed and its effect on results and computing time is discussed and concluded that a value of 60 m/s was suitable for simulating vascular biomechanical problems. Two methods were used: foot-to-foot measurement of velocity waveforms and slope of the regression line of the wall radial deflection wave peaks throughout a contour plot. Both methods showed coincident results. Results were approximately 6% less than those calculated from the Moens-Korteweg equation. The proposed method was able to describe the increase in the stiffness of the walls of large human arteries via the PWV estimates.

## 1. Introduction

Computational analysis of cardiovascular problems incorporating FSI is a challenging problem. Detailed analysis of the blood flow field and artery wall behavior can assist in clinicians' assessment of vascular diseases [1]. The first person to investigate a formula for the velocity of pressure waves in a thin elastic tube was Young [2] in 1808. Womersley [3] investigated the dynamic response of a tube with a sinusoidal flow and defined an analytical solution for the flow domain.

In this study, we investigated the propagation of a pulse wave through an elastic vessel. This application is of clinical relevance as PWV measurements are currently considered to be the clinical gold-standard measure of arterial stiffness [4]. PWV is typically a disturbance's propagation speed through a vessel resulting from the flow pressure. As blood is an almost-incompressible fluid [5–7], the finite PWV is mainly the result of the FSI between the local pressure of the blood on the vessel wall and the resultant wall deformation it causes.

To validate the obtained results, we used the same model used by Kuntz et al. [8] and Penrose and Staples [9] who validated their simulation, conducted with ANSYS-CFX commercial software, with the theoretical results obtained by the Moens-Korteweg equation [10]. Moatamedi et al. [11] and Souli et al. [12] used the same model in their study and validated their simulation, conducted with LS-DYNA commercial package, with the results obtained by the Moens-Korteweg equation.

Shahmirzadi et al. [13] validated their work conducted with ABAQUS explicit solver with the Moens-Korteweg equation, but they used a different model. Dias et al. [14] implemented their model using the commercial code RADIOSS (Altair Engineering) to investigate the effect of both elasticity and wall thickness on PWV through a long elastic tube.

## 2. Flow in an Elastic Vessel

The transient progression of a pressure pulse through a tube has been investigated by many researchers over the years. A good review of this research is available [17]. The first work on wave propagation in an elastic tube was presented by Moens and Korteweg at the end of the 19th century [10]. It is based on Newton's work on the speed of sound in air. Taking *E* as the wall's Young's Modulus, *h* as the thickness of the wall, *R* as the inner radius, and *ρ* as the fluid density and relating the change in radius to the applied pressure, the wave speed (*c*_0_) can be written as

(1) $$\begin{array}{c} c_{0}=\sqrt{\frac{E\cdot h}{2R\cdot \rho }}. \end{array}$$

Errors resulting from the thin tube assumption can be compensated by using the Bergel correction [18], which accounts for the thickness through Poisson's ratio (*υ*). The difference between the modified wave speed (*c*′) and the original one (*c*_0_) is calculated by the following equation:

(2) c′c02=2−γ2−2γ1−υ−2υ2+γ21−υ−υ2−2υ2,

where *γ* is the ratio of the wall thickness and tube outer radius. Simplifying this expression and incorporating it into (1), the modified wave speed (*c*′) becomes

(3) $$\begin{array}{c} c^{\prime }=\sqrt{\frac{E\cdot h}{2R\cdot \rho \left(1-\upsilon^{2}\right)}}. \end{array}$$

## 3. Numerical Modeling

**Figure 1:**
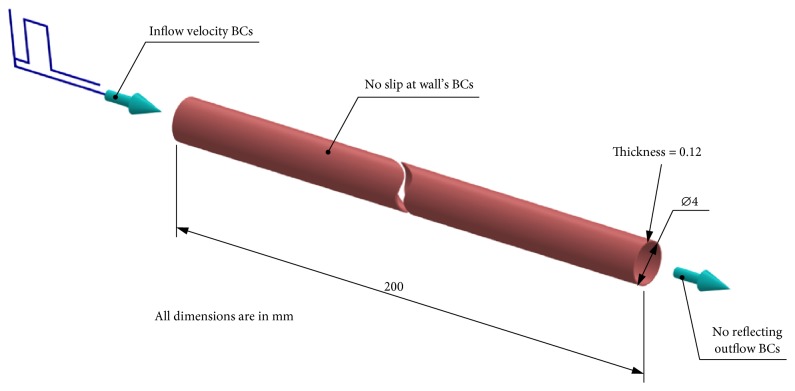
The long elastic tube model and boundary conditions.

The numerical setup used for this three-dimensional fluid structure interaction study was based on a tube with an internal diameter of 4 mm and a wall thickness of 0.12 mm as shown in Figure 1. The length of the model was set as 100 times as long as the internal radius of the tube to be long enough as per the Moens-Korteweg equation's condition. The vessel wall was considered as elastic with a density of 1075 kg/m^3^, a Poisson's ratio of 0.45, and an initial Young's modulus (*E*) of 3 MPa. These values are well representative of a blood vessel's physiological state. The blood material was modeled with a Newtonian incompressible equation of state (EOS) which related the density of the blood (*ρ*) to the external pressure *P* and the sound speed (*c*) according to the following equation:

(4) $$\begin{array}{c} \rho =\rho_{o}+\frac{P}{c}, \end{array}$$

where *ρ*_*o*_ is the initial blood density. This constitutive material law gives slight compressibility to the blood, which improves the stability and convergence of our computation. Thus, the higher the sound speed the higher the incompressibility of the fluid. The initial blood density (*ρ*_*o*_) was set to 1000 kg/m^3^ and dynamic viscosity was 0.001 Pa·s. The sound speed *c*_*o*_ was set to 60 m/s according to a separate study, where a range of sound speeds were tested, ranging from 15 m/s to the real value of 1460 m/s. It was found that a sound speed of 60 m/s provided reasonable results and incurred less computational cost. Higher values yielded similar accuracy but with relatively high computational cost. Lower values such as 15 m/s led to underestimation in the results. We thus concluded that a value of 60 m/s was practical for simulating vascular biomechanical problems.

From (4), it can be seen that the bulk modulus *K* of the blood can differ by more than 10^3^ more than the arterial wall's Young's modulus (*E*). Materials with elastic modulus differing by 10^3^ or more are not practicable for the FSI study with the current available modeling techniques as noted by Moatamedi et al. [11].

(5) $$\begin{array}{c} K=\rho {c_{o}}^{2}. \end{array}$$

**Figure 2:**
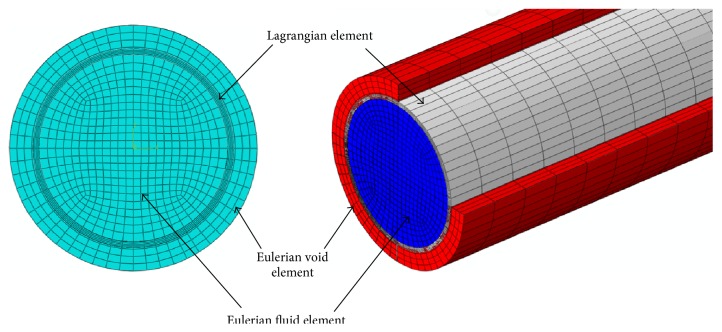
Meshed parts of model.

**Figure 3:**
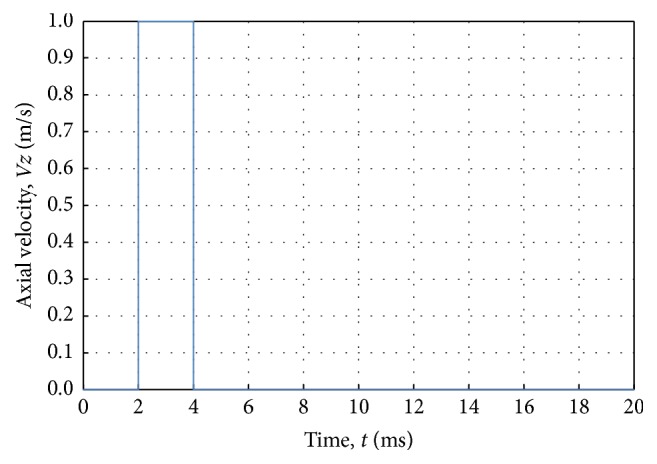
Inlet boundary conditions; a velocity pulse in the axial direction.

The Lagrangian solid elements were imposed inside the Eulerian elements, and the void elements were added to the outer surface of the Lagrangian element so that the deflected artery walls were always surrounded by Eulerian mesh during the dynamic analysis as shown in Figure 2. The problem was set up with 141400 linear hexahedra elements assigned for the fluid and 44800 linear hexahedra elements assigned for the artery, with four layers of elements through the radial thickness. The elements' thickness along the longitudinal direction was 1 mm. All translational degrees of freedom constraints were applied on the inlet and exit sections of the vessel wall. A single rectangular pulse of 1 m/s over a period of 2 ms (Reynolds number = 4000) as shown in Figure 3 was applied at the tube inlet to serve as the driving force to produce a propagating wave. A no-slip FSI was defined between the wall and the fluid. A nonreflecting boundary condition was pointed at the tube outlet to reduce the wave reflection due to the truncation of the computational domain (see (6)).

Grote and Keller [19, 20] derived two sets of nonreflecting boundary conditions for the wave equation:

(6) B1ϕx,t+1R∑n=1∞ ∑m=−nnen·znmtYnmθ,φ=0B2ϕx,t+1R∑n=2∞ ∑m=−nne^n·znmtYnmθ,φ=0,

where *ϕ*(*x*, *t*) is the solution to the scalar wave equation, *R* is the radius of a spherical artificial boundary *Г*, *Y*_*nm*_ are spherical harmonics, *e*_*n*_ and ${\hat{e}}_{n}$ are vectors of coefficients, and *z*_*nm*_(*t*) are solutions to a first-order system of ordinary differential equations driven by the radial harmonics *ϕ*_*nm*_ = (*ϕ*, *Y*_*n*_)*Г*. The summation over the series in (6) may be considered as extensions of the local *B*_1_ and *B*_2_ operators, respectively, of Bayliss and Turkel [21]. In computation, the sum over *n* in (6) is truncated at an arbitrary value *N* ≥ 1 and *N* ≥ 2, respectively. Both boundary conditions were exact for modes *n* ≤ *N*.

## 4. Wave Propagation

**Figure 4:**
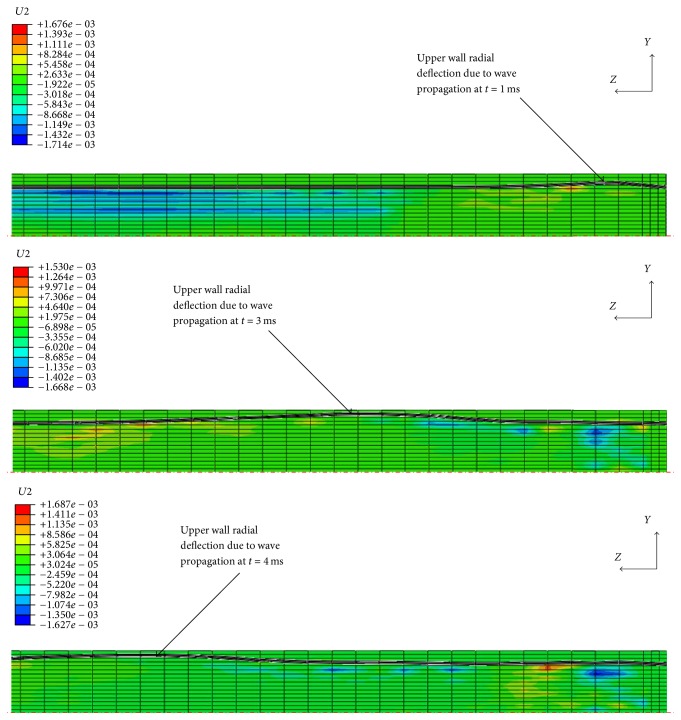
Three different time frames of radial disturbance of tube wall because of wave propagation. The displacement of the structure has been magnified by factor of 5. The unit of the scale is mm.

The simulation ran with a 20 ms explicit step time for a Young's moduli range of 3, 2, 1, 0.5, and 0.1 MPa assigned to the tube material. Using a Dell computer with an Intel® Xeon® CPU running at 2.8 GHz with 12 processors and 24 GB RAM, it took approximately 13 h to complete each calculation.  Figure 4 shows the radial disturbance of the tube wall because of wave propagation in three different time frames.

## 5. Velocity Waveforms

**Figure 5:**
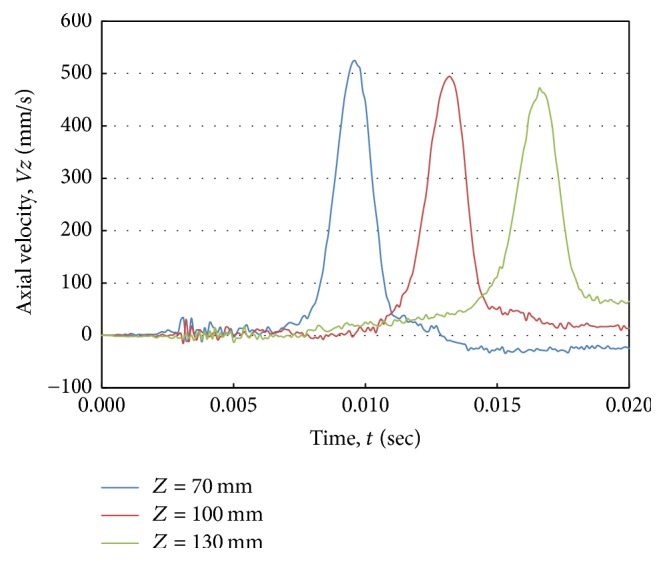
Axial velocity waveforms at different positions along the tube center line for an artery with Young's modulus (*E*) of 3.0 MPa.

Figure 5 shows the velocity waveforms at different locations along the tube center line typically at distances 70, 100, and 130 mm from the tube inlet. The waveforms were plotted from the axial velocity component (*Vz*). The fluctuations of the velocity curve are a result of FSI. The peaks of the waveforms decreased with simulation time and shifted in the flow direction because of the damping effect.

## 6. Pulse Wave Velocity Assessment

Two different methods were used to assist the PWV: foot-to-foot measuring of velocity waveforms and slope of the regression line of the wall radial deflection wave peaks throughout a contour plot.

### 6.1. Foot-to-Foot Velocity Waveform Method

**Figure 6:**
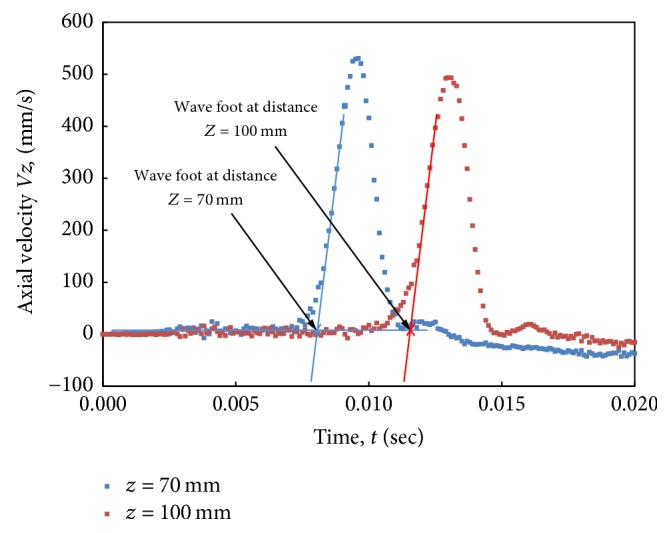
Arrival time for two progressive waveforms indicated by foot-to-foot method.

The PWV was calculated from the time delay of each waveform relative to its preceding waveform. The waveforms were plotted for equal-spaced intervals along the tube center line. To measure the time interval between two sites, we used the foot-to-foot method [22]. As shown in Figure 6, a best fit straight line was fitted to the velocity ascending points between 20% and 80% of the maximum. The pulse arrival time was defined as the point where the line intersected the baseline. The pulse arrival time was determined for two progressive waveforms. PWV was calculated from the distance between the two positions (*l*), where the axial velocity was plotted, divided by the difference in arrival time (Δ*t*):

(7) $$\begin{array}{c} \text{PWV}=\frac{l}{\Delta t}. \end{array}$$

We take the sites at distances 70, 100, and 130 mm from the tube inlet to be far away from the tube ends, where wave reflections are expected due to fixed boundary conditions at those ends.

### 6.2. Slope of Regression Line of Wall Radial Deflection Wave Peaks

**Figure 7:**
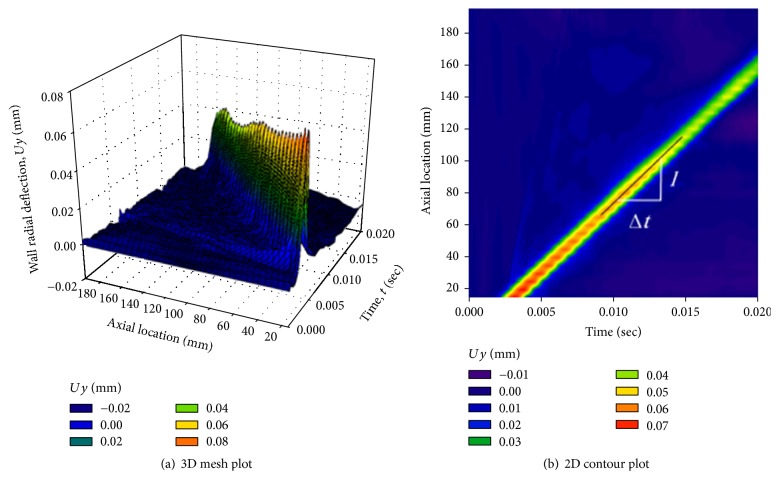
Model with Young's modulus, *E* = 3.0 MPa. (a) 3D mesh plot of the wall radial deflection against temporal and spatial coordinates showing the peaks of the forward waves. (b) 2D contour plot of the wall radial deflection. The slope of the peaks regression line indicates the PWV.

The wall radial deflection at equally spaced locations along the tube center line was plotted against time and is shown as a 3D mesh plot in Figure 7(a), where the peaks of the forward waves are shown.  Figure 7(b) shows the contour plot of the wall radial deflection against time and axial locations. The color coding clearly shows the slope of the waves' peaks through the forward direction. The slope of the regression line represents the value of the PWV. Both methods gave coincident results for the PWV.

## 7. Validation of Numerical Simulation

### 7.1. Validation with the Idealized Theory

**Figure 8:**
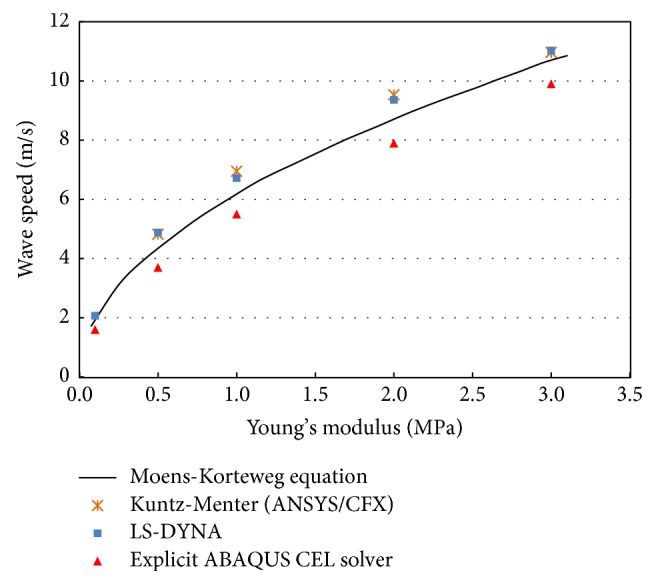
PWV as a function of Young's modulus: comparison of numerical results conducted with different commercial packages and values calculated from the Moens-Korteweg equation.

The obtained results were plotted with the values calculated from the modified Moens-Korteweg equation, (3), as well as the results obtained by ANSYS-CFX and LS-DYNA as shown in Figure 8. These results were computed for different Young's moduli for the tube material. The obtained results corresponded well with the idealized theory with underestimation depending on the value of a wall's Young's modulus.

### 7.2. Validation with In Vivo Measurements of PWV

**Figure 9:**
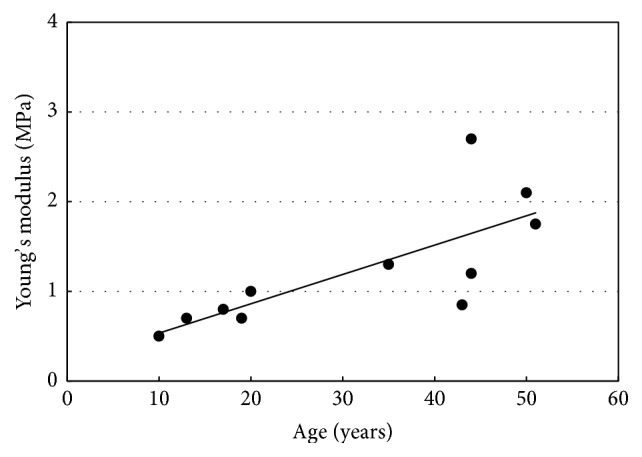
Effect of ageing on elastic properties of arteries; incremental Young's modulus plotted against age for normal human aorta at a pressure of 100 mm Hg [15].

**Figure 10:**
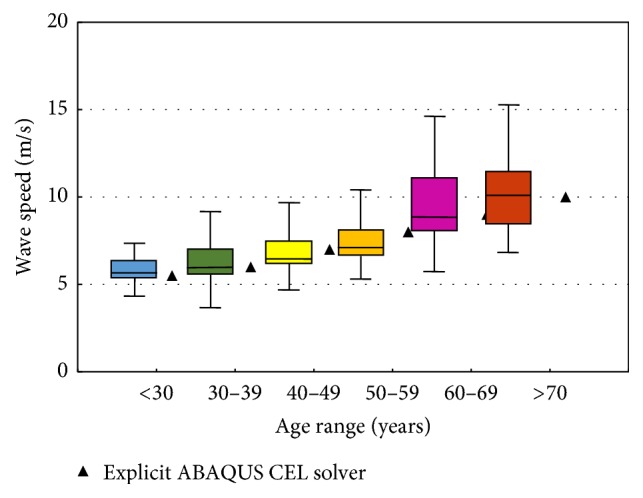
Normal values for PWV (average according to age (1455 subjects); boxes contain 50% of the data and bars contain the remainder; horizontal lines indicate medians) [16] plotted with the corresponding values obtained from our approach.

Figure 9 shows the effect of ageing on elastic properties of arteries, where incremental Young's modulus is plotted against age for normal human aorta at a pressure of 100 mm Hg according to the work of Caro et al. [15]. We use our approach to model the PWV corresponding to each age interval using those values for Young's modulus. The obtained results are plotted with in vivo PWV values obtained from literature [16] as shown in Figure 10. The graph shows that the obtained results corresponded well with the normal values for in vivo PWV.

## 8. Discussion

Figure 8 shows that the PWV is proportional to wall stiffness. The obtained results are slightly underestimated compared to the basic theory. The underestimation is attributed to two basic reasons. First, waves were reflected because of the finite tube length and fixed boundary conditions, which pointed at the tube ends. Second, the scaling down of the speed of sound added some compressibility to the fluid resulting in significant damping, which played a role in slowing the wave propagation speed. The underestimation difference increased with increases in wall stiffness. Therefore, we kept underestimation at a minimum of 7% corresponding to Young's modulus *E* = 3.0 MPa by using a “long enough” tube length with a tube length to radius ratio, *l*/*r*, of 100 and by applying nonreflecting boundary conditions at the tube outlet. Scaling down the speed of sound to 60 m/s provided a reasonable computational cost without affecting the accuracy of the obtained results. However, the results obtained from other models such as those of Dias et al. [14] and Shahmirzadi and Konofagou [23] who implemented their work with the commercial code RADIOSS (Altair Engineering) and ABAQUS (SIMULIA, USA), respectively, also show underestimation in most cases.

## 9. Conclusion

In this study, a basic computational scheme for a strongly coupled FSI in an elastic artery was developed and validated with basic theory. Qualitative agreement was obtained, indicating that this computational method for PWV analysis is accurate enough to evaluate its value with accepted accuracy. The scaling down of sound speed has a significant effect on results convergence and computation cost, and we conclude that a value of 60 m/s is reasonable enough for solving vascular biomechanical problems. On the other side, the PWV values obtained from our new approach corresponded well with in vivo reference values published in literatures [15, 16].
